# Mechanism of action of Houttuynia cordata extract and its application in animal production

**DOI:** 10.3389/fvets.2026.1834144

**Published:** 2026-05-26

**Authors:** Duhan Xu, Hanyu Wang, Shengyong Lu, Maosheng Cao, Wen Tang, Shengchang Chen, Shan Yang, Shihui Mei, Mingming Zhu, Binbin Na, Xin Liu, Yao Lei, Xue Li, Qiming Cheng, Chao Chen

**Affiliations:** 1 College of Animal Science, Guizhou University, Guiyang, China; 2 Guizhou Provincial Center for Animal Disease Prevention and Control, Guiyang, China; 3 Key Laboratory of Animal Genetics, Breeding and Reproduction in The Plateau Mountainous Region, Ministry of Education, Guizhou University, Guiyang, China; 4 Key Laboratory of Animal Genetics, Breeding and Reproduction, Guiyang, China

**Keywords:** anti-inflammatory, antioxidant, feed additive, Houttuynia cordata extract, immune regulation

## Abstract

The global phasing-out of antibiotic growth promoters (AGPs) has catalyzed an urgent search for efficacious and sustainable alternatives to maintain livestock health and productivity. *Houttuynia cordata Thunb*. (HC), a traditional pharmacophagous herb has emerged, as a promising candidate due to its pleiotropic bioactive profile. This review outlines the molecular mechanisms and clinical efficacy of HC extracts (HCE) as multifunctional feed additives. We elucidate that HCE, rich in volatile oils, flavonoids, and polysaccharides, systemically modulates host physiology by activating the Nrf2/HO-1 antioxidant signaling axis while concurrently suppressing hyper-inflammation via the inhibition of TLR4/NF-κB and NLRP3 inflammasome pathways. Crucially, HCE reinforces epithelial integrity across various biological barriers (intestinal and mammary) through the upregulation of tight junction proteins. Across diverse production systems, HCE has significant efficacy under experimental conditions. In ruminants, it alleviates heat stress-induced redox imbalance and mitigates mastitis. In monogastric species, it enhances viral resistance (notably against PRRSV) and optimizes meat quality. In aquaculture, it fortifies non-specific mucosal immunity against viral challenges. Despite technical hurdles regarding component stability and standardized quality control, HCE represents a transformative, green nutritional strategy. Advancements in microencapsulation and nano-emulsification are poised to catalyze the industrial integration of HCE, positioning it as a cornerstone of sustainable animal agriculture in the post-antibiotic era.

## Graphical Abstract

**Figure:**
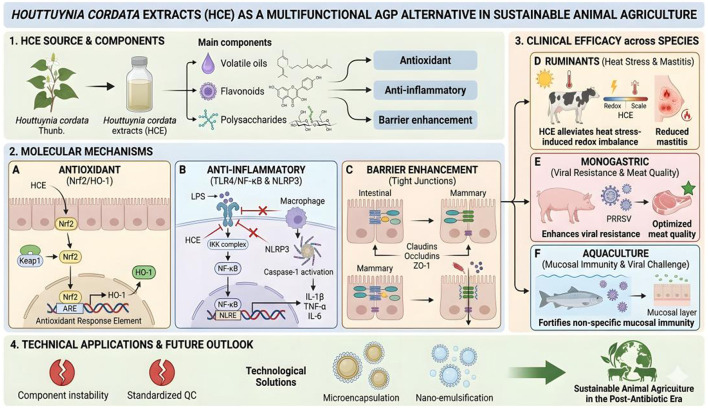
(1) HCE source and bioactive components: HCE is rich in core bioactive components, including volatile oils, flavonoids, and polysaccharides. (2) Molecular mechanisms (A–C): (A) Antioxidant mechanism: HCE promotes the dissociation of Nrf2 from Keap1 and its subsequent nuclear translocation, where it binds to the antioxidant response element (ARE) and upregulates the expression of downstream antioxidant genes such as heme oxygenase-1 (HO-1). (B) Anti-inflammatory mechanism: HCE blocks LPS-activated TLR4 signaling and inhibits the IKK complex, thereby preventing NF-κB nuclear translocation. It also suppresses NLRP3 inflammasome assembly and Caspase-1 activation in macrophages, reducing the release of pro-inflammatory cytokines including IL-1β, TNF-α, and IL-6. (C) Barrier-enhancing mechanism: HCE significantly upregulates the expression and assembly of tight junction proteins in intestinal and mammary epithelial cells, thereby strengthening epithelial barrier integrity. (3) Cross-species clinical efficacy (D–F): (D) ruminants, (E) monogastric animals, and (F) aquaculture. (4) Technological applications and future perspectives.

## 1 Introduction

The intensification of global livestock production has presented the industry with significant challenges. Historically, antibiotics used as growth promoters (AGPs) have played a critical role in disease prevention and enhancing feed conversion efficiency (1). However, this reliance has precipitated a serious public health crisis, encompassing the cross-species transmission of antimicrobial resistance (AMR), drug residues in animal-derived foods, and ecological microbiome disruption (2). To address this global threat, the European Union implemented a full ban on the use of antibiotics as feed additives in 2006 (3). Similarly, China, as the world's largest livestock producer, enacted the Ministry of Agriculture and Rural Affairs Announcement No. 194, initiating a comprehensive ban on in-feed antibiotics starting in 2020. This regulatory shift has created a pressing need within the industry to identify effective, safe, and green natural plant-based alternatives to antibiotics. Among numerous candidate plants, Houttuynia cordata (HC) stands out due to its remarkable medicinal value and wide geographical distribution (4). Commonly known as fishwort or “Zhe'ergen,” HC belongs to the Saururaceae family and boasts a millennia-long history of medicinal and culinary use in East Asia. In traditional medicine, it is renowned for its properties of “clearing heat, detoxifying, reducing swelling, draining pus, and promoting diuresis,” often referred to as a “natural antibiotic”(5). Modern pharmacological research further reveals that HC is not merely a simple antimicrobial agent but rather a bioactive complex with multi-target regulatory functions. Listed in the Catalogue of Feed Materials by China's Ministry of Agriculture and Rural Affairs, HC's combined anti-inflammatory, antiviral, antioxidant, and immunomodulatory activities make it an ideal candidate for replacing antibiotics in livestock, poultry, and aquaculture (6). This review aims to summarize the bioactive components of HCE, elucidate its molecular mechanisms of action involving key signaling pathways such as Nrf2/HO-1 and TLR4/NF-κB, and comprehensively evaluate its application efficacy and safety in swine, poultry, ruminant, and aquatic animal production. The goal is to provide a scientific foundation for the further development and rational application of HCE in modern animal husbandry.

### Search strategy and selection criteria

Relevant literature was identified by searching electronic databases including PubMed, Web of Science, Scopus, and Google Scholar for articles published between 2016 and 2026. Keywords used for the search included “Houttuynia cordata,” “HCE,” “phytogenic feed additives,” “antibiotic alternatives,” “growth performance,” and “livestock”. The inclusion criteria focused on peer-reviewed original research articles, meta-analyses, and systematic reviews specifically addressing the molecular mechanisms and production applications of HCE in ruminants, monogastrics, and aquaculture.

## 2 Bioactive components

The chemical composition of Houttuynia cordata is diverse, primarily consisting of four major classes: volatile oils (essential oils), flavonoids, alkaloids, and polysaccharides. These components act synergistically to form the pharmacological basis of HCE.

### 2.1 Volatile oils (essential oils)

Volatile oils are the most characteristic constituents of HC, imparting its distinctive odor and serving as the primary basis for its potent antibacterial activity. Gas chromatography-mass spectrometry (GC-MS) analyses reveal that HC volatile oil contains dozens of compounds. Key constituents include houttuynin (decanoyl acetaldehyde), the principal antibacterial component exhibiting significant inhibitory effects against various pathogenic bacteria such as *Staphylococcus aureus* and *Escherichia coli* (7). However, houttuynin is chemically unstable and prone to oxidation and polymerization. Therefore, its stable sodium derivative, sodium houttuyfonate, is commonly used in practical applications. Furthermore, 2-undecanone not only possesses broad-spectrum antibacterial activity but also demonstrates notable anti-inflammatory and anti-tumor potential by modulating cellular signaling pathways to inhibit the release of inflammatory factors (8). Other terpenoids, such as lauric aldehyde, β-myrcene, and α-pinene, also contribute synergistically to enhance the overall bioactivity of the volatile oil (9).

### 2.2 Flavonoids

Flavonoids represent the core active constituents responsible for HC's antioxidant, antiviral, and immunomodulatory effects. These compounds act through multiple synergistic mechanisms, forming the core of HCE's pharmacological profile (10). Major components include quercitrin, often used as a key marker for quality control. Quercitrin possesses both antioxidant and antiviral capacities, effectively inhibiting the adsorption and replication processes of enveloped viruses (11). Quercetin, the aglycone form of quercitrin, exhibits higher bioavailability. It is not only a potent free radical scavenger that directly neutralizes reactive oxygen species but also a significant activator of the Nrf2 antioxidant pathway (12). Quercetin can upregulate the expression of various Phase II detoxification enzymes and antioxidant proteins within cells, thereby enhancing cellular defense against oxidative damage (13). Additionally, glycosidic flavonoids like hyperoside and rutin, upon hydrolysis and metabolism *in vivo*, can inhibit the production of pro-inflammatory cytokines by modulating inflammatory signaling pathways such as NF-κB. This action alleviates tissue inflammation and also demonstrates protective effects on vascular endothelial function, aiding in the maintenance of normal microcirculation (14). Research indicates that synergistic effects likely exist among these flavonoid components, with the composite bioactivity of the mixture often surpassing that of individual compounds. This further underscores the comprehensive advantage of HC as a multi-component natural product in regulating immune balance and counteracting oxidative stress (10).

### 2.3 Alkaloids and polysaccharides

In addition to volatile oils and flavonoids, the alkaloid and polysaccharide fractions of HC constitute important material bases for its multi-dimensional bioactivity. Among the alkaloids, the primary types are aristolactams and aporphine alkaloids (15). Although certain aristolactam components from some plants in the Aristolochiaceae family have garnered attention due to nephrotoxicity, the specific alkaloids found in HC (e.g., Aristolactam BII) differ significantly in chemical structure, content, and metabolic pathways (16). Multiple toxicological assessments, including acute toxicity, subacute toxicity, and mutagenicity tests, have confirmed that within the standard dosage range for feed additives (typically 0.05%−0.2% of feed), these HC-derived alkaloids do not cause significant adverse effects on liver/kidney function or histopathology in experimental animals. This provides crucial scientific support for the compliant use of HC in feed (17).

HC polysaccharides, as high-molecular-weight natural polymers, are widely regarded as potent immunoenhancers. Their immunomodulatory mechanisms operate at multiple levels: at the cellular immunity level, they can directly or indirectly promote the proliferation and differentiation of splenic lymphocytes and thymic T-lymphocytes, while enhancing macrophage phagocytic activity and nitric oxide release (18), at the humoral immunity level, they can assist in elevating antibody (e.g., IgG, IgA) production levels (19). HC polysaccharides primarily function by activating pattern recognition receptors (e.g., Toll-like receptors) on macrophage surfaces, subsequently regulating downstream signaling pathways such as NF-κB and MAPK. This leads to the secretion of cytokines like interleukin-2 (IL-2) and interferon-gamma (IFN-γ), thereby bolstering the body's non-specific immune defense and disease resistance (20). Furthermore, structural characteristics of polysaccharides, including molecular weight, degree of branching, and monosaccharide composition, are closely related to their immunomodulatory activity (21). We have summarized the components and functions of HCE in Table 1.

**Table 1:** Detailed phytochemical composition and multi-target molecular mechanisms of HCE.

Chemical class	Key bioactive compounds (markers)	Typical content/ characteristics	Molecular targets & pathways	Primary biological functions
Volatile oils	Houttuynin (decanoyl acetaldehyde), 2-undecanone (MNK)	0.1%−0.8% of fresh weight; Houttuynin is the primary marker	Inhibition of TLR4/NF-κB signaling and NLRP3 inflammasome activation	Broad-spectrum antibacterial (S. aureus, E. coli), anti-inflammatory, and antifungal
Flavonoids	Quercitrin, quercetin, hyperoside, rutin	0.5%−1.5% of dry matter; Quercitrin is the standard marker	Activation of Nrf2/HO-1 antioxidant axis; inhibition of viral 3CLpro and RdRp enzymes	Redox homeostasis, antiviral (PRRSV, PEDV, IBV), and vascular protection
Polysaccharides	Houttuynia cordata polysaccharide (HCP)	High-molecular-weight polymers; ~2.5%−5.0% in extracts	Activation of toll-like receptors (TLRs); modulation of MAPK pathway and cytokine secretion	Immunoenhancement, promotion of lymphocyte proliferation, and IgG/IgA elevation
Alkaloids	Aristolactam BII, aporphine alkaloids	Trace levels; verified safe within standard inclusion (0.05%−0.2%)	Modulation of pro-inflammatory mediators (iNOS, COX-2) and cellular metabolism	Synergistic anti-inflammatory regulation and tissue protection

## 3 Functional mechanisms of Houttuynia cordata extract

The efficacy of HCE as an AGP alternative in livestock production stems not merely from direct bactericidal effects but, more importantly, from its ability to systemically modulate host physiology. Recent literature elucidates the sophisticated molecular regulatory mechanisms of HCE in antioxidant defense, anti-inflammation, intestinal barrier repair, and antiviral activity.

### 3.1 Antioxidant defense

**Figure 1:**
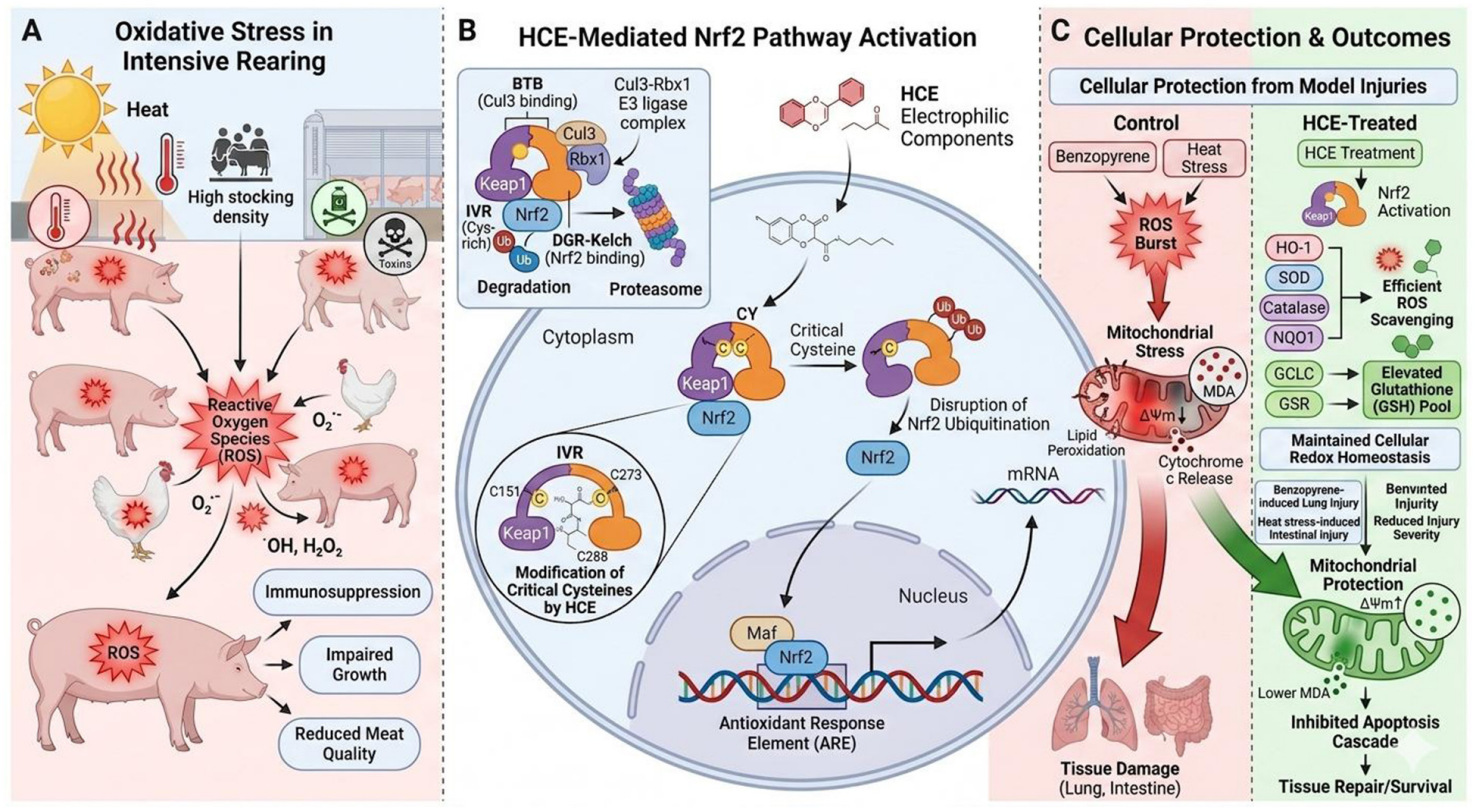
Antioxidant mechanism of HCE. **(A)** Oxidative stress induced by intensive rearing (heat, high stocking density) leads to excessive ROS production, resulting in immunosuppression, impaired growth, and reduced meat quality. **(B)** HCE activates the Nrf2 pathway by interfering with Keap1-Nrf2 binding, preventing Nrf2 ubiquitination and proteasomal degradation, allowing Nrf2 translocation to the nucleus. **(C)** In the control condition (left), model injuries (e.g., benzopyrene, ROS burst) cause mitochondrial stress, lipid peroxidation (MDA), and tissue damage. In HCE-treated condition (right), HCE upregulates antioxidant enzymes (HO-1, SOD, Catalase, NQO1, GCLC, GSR), maintains redox homeostasis, and enhances ARE-mediated cytoprotection, thereby alleviating tissue damage.

Oxidative stress is a central factor contributing to immunosuppression, impaired growth, and reduced meat quality in intensively reared animals (Figure 1). HCE, particularly its rich flavonoid content and components like 2-undecanone, has been demonstrated to be a potent activator of the Nrf2 (Nuclear factor erythroid 2–related factor 2) signaling pathway, systematically initiating the body's endogenous antioxidant defense system (22). Under normal physiological conditions, the transcription factor Nrf2 is sequestered in the cytoplasm by its inhibitor protein Keap1 and maintained at low levels via ubiquitin-mediated degradation (23). When cells are stimulated by electrophilic active components in HCE, these components can modify critical cysteine residues on the Keap1 protein, inducing a conformational change in Keap1 that leads to Nrf2 release (24). Subsequently, Nrf2 translocates to the nucleus, binds to the antioxidant response element (ARE), and initiates the transcription of a battery of downstream antioxidant enzyme genes. These include heme oxygenase-1 (HO-1), superoxide dismutase (SOD), catalase (25), and NAD(P)H quinone dehydrogenase 1 (NQO1) (26). This transcriptional activation not only directly scavenges intracellular reactive oxygen species but also helps maintain cellular redox homeostasis by elevating glutathione (GSH) levels (27). Studies show that in models of benzopyrene-induced lung injury and heat stress-induced intestinal injury, HCE, via Nrf2 pathway activation, significantly reduces the accumulation of the lipid peroxidation end product malondialdehyde (MDA), protects mitochondrial functional integrity, and thereby effectively inhibits the apoptosis cascade (28).

### 3.2 Anti-inflammatory regulation

**Figure 2:**
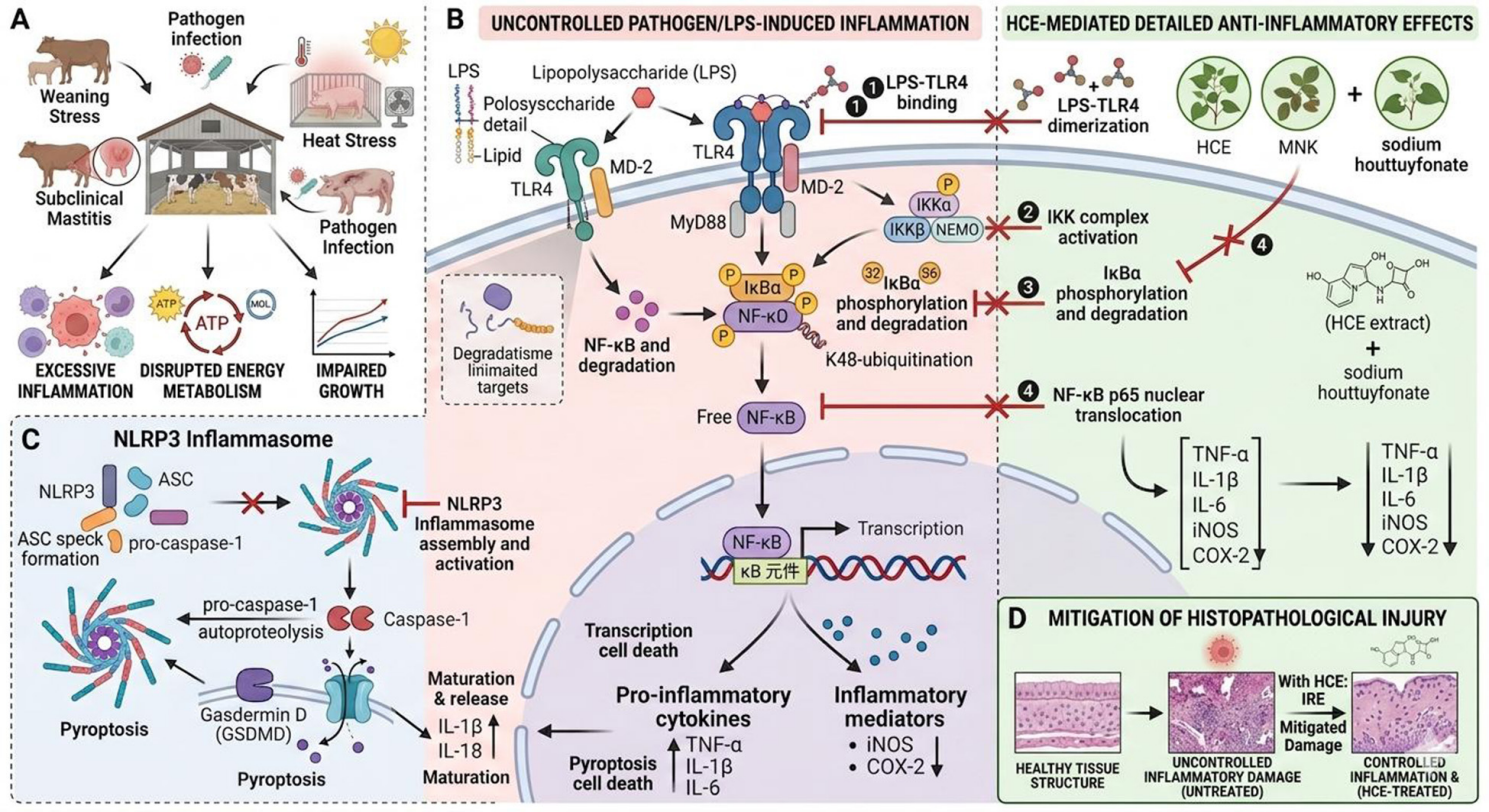
Anti-inflammatory regulatory mechanism of HCE. **(A)** Stressors in the rearing environment and their negative effects: Common stress factors in livestock production (e.g., weaning stress, subclinical mastitis, pathogen infection, and heat stress) lead to excessive inflammation, disruption of energy metabolism, and ultimately impaired growth performance. **(B)** HCE-mediated anti-inflammatory mechanism targeting the TLR4/NF-κB signaling pathway: The left side of the figure shows the uncontrolled LPS-induced inflammatory pathway; the right side shows the specific intervention sites of HCE, MNK, and sodium houttuynonate. **(C)** Targeting the NLRP3 inflammasome and pyroptosis: Inhibition of NLRP3 inflammasome assembly and activation prevents the autocatalytic cleavage of pro-caspase-1, thereby blocking Gasdermin D (GSDMD)-mediated pyroptosis and reducing the maturation and release of IL-1β and IL-18. **(D)** Amelioration of histopathological damage: Representative tissue section comparisons show, in order, healthy tissue structure, severe uncontrolled inflammatory damage in the untreated group, and the tissue morphology after HCE treatment with controlled inflammation and significantly alleviated pathological damage.

Inflammation is a vital physiological process for defending against pathogen infection (Figure 2). However, excessive or chronic inflammation (e.g., from weaning stress, subclinical mastitis, heat stress) disrupts energy metabolism and significantly impairs animal growth (29). HCE exhibits multi-target anti-inflammatory effects, inhibiting the inflammatory cascade upstream at the signaling pathway level. Within the TLR4/NF-κB pathway, HCE and its active constituents (e.g., MNK, sodium houttuyfonate) can interfere with the binding of lipopolysaccharide (LPS) to Toll-like receptor 4 (TLR4) and its dimerization. They also inhibit the phosphorylation and degradation of the downstream inhibitor IκBα, thereby blocking the nuclear translocation of the NF-κB p65 subunit. This ultimately leads to a marked reduction in the expression of pro-inflammatory cytokines such as TNF-α, IL-1β, and IL-6, as well as inflammatory mediators like inducible nitric oxide synthase (iNOS) and cyclooxygenase-2 (COX-2) (30). Furthermore, HCE can inhibit the assembly and activation of the NLRP3 inflammasome, preventing caspase-1-mediated cleavage of gasdermin D (GSDMD). This action suppresses pyroptosis and the maturation/release of IL-1β and IL-18, thereby alleviating tissue inflammatory damage (31). This dual inhibition of the TLR4/NF-κB and NLRP3 signaling axes underscores the significant application value of HCE in controlling infectious inflammation and mitigating histopathological injury.

### 3.3 Barrier repair

**Figure 3:**
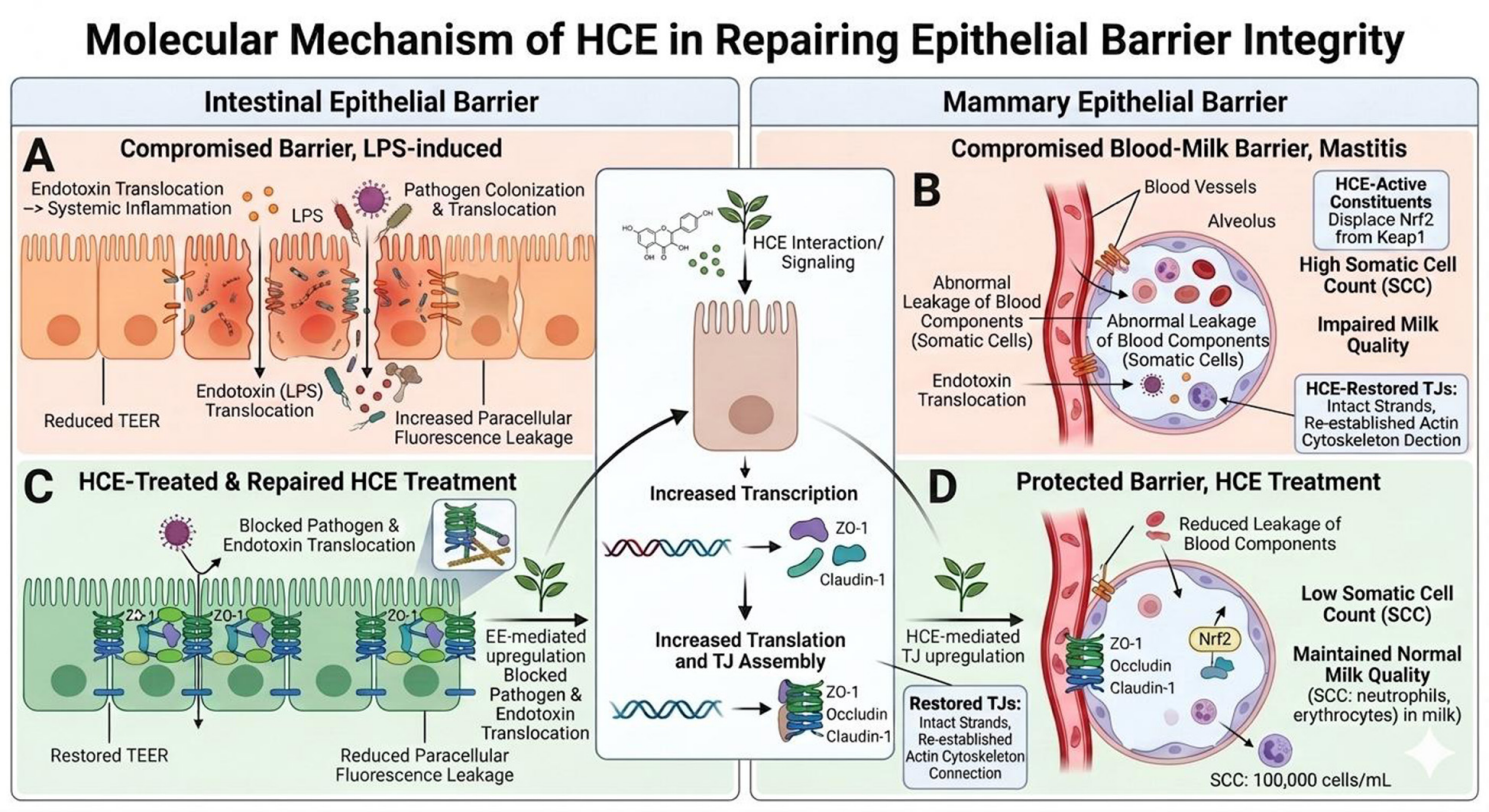
Barrier repair mechanism of HCE. **(A)** LPS-induced impairment of the intestinal epithelial barrier: Under pathogen stimulation such as lipopolysaccharide (LPS), the intestinal epithelial barrier is disrupted, leading to decreased transepithelial electrical resistance (TEER) and increased paracellular permeability (e.g., enhanced leakage of fluorescent tracers). This facilitates pathogen colonization and endotoxin translocation, ultimately triggering systemic inflammation. **(B)** Mastitis-induced impairment of the blood-milk barrier: Under the pathological condition of mastitis, the integrity of the blood-milk barrier is compromised, resulting in abnormal leakage of blood components (e.g., somatic cells) into the mammary alveoli. This leads to a significant increase in milk somatic cell count (SCC) and a marked decline in milk quality, accompanied by translocation of endotoxins across the barrier. **(C)** HCE-mediated repair of the intestinal epithelial barrier: Following HCE treatment, tight junctions (TJs) between intestinal epithelial cells are reassembled and restored. TEER returns to normal levels, paracellular leakage is effectively reduced, and the translocation of pathogens and endotoxins is successfully blocked. **(D)** HCE-mediated protection of the mammary blood-milk barrier: The active components of HCE upregulate the expression of tight junction proteins and reconstruct the actin cytoskeleton connections through signaling pathways such as promoting the dissociation of Nrf2 from Keap1.

The integrity of the intestinal and mammary epithelial barriers serves as a structural defense against pathogen colonization and translocation (Figure 3). Compromised barrier function leads to endotoxin translocation and systemic inflammation (32). At the molecular level, HCE has been shown to upregulate the expression of key tight junction proteins in epithelial cells, including zonula occludens-1 (ZO-1), occludin, and claudin-1. These proteins collectively regulate paracellular permeability, constituting a critical physical barrier (33). In LPS-induced intestinal injury models, HCE treatment significantly increased the transcriptional and translational levels of ZO-1 and occludin, restored transepithelial electrical resistance (TEER), and effectively blocked the paracellular leakage of fluorescence-labeled macromolecules (34). In bovine mastitis, HCE similarly demonstrates a protective effect on blood-milk barrier integrity. By upregulating tight junction protein expression, it reduces the abnormal leakage of blood components into milk, thereby helping to lower somatic cell count (SCC) and maintain normal milk quality (35).

### 3.4 Antiviral activity

**Figure 4:**
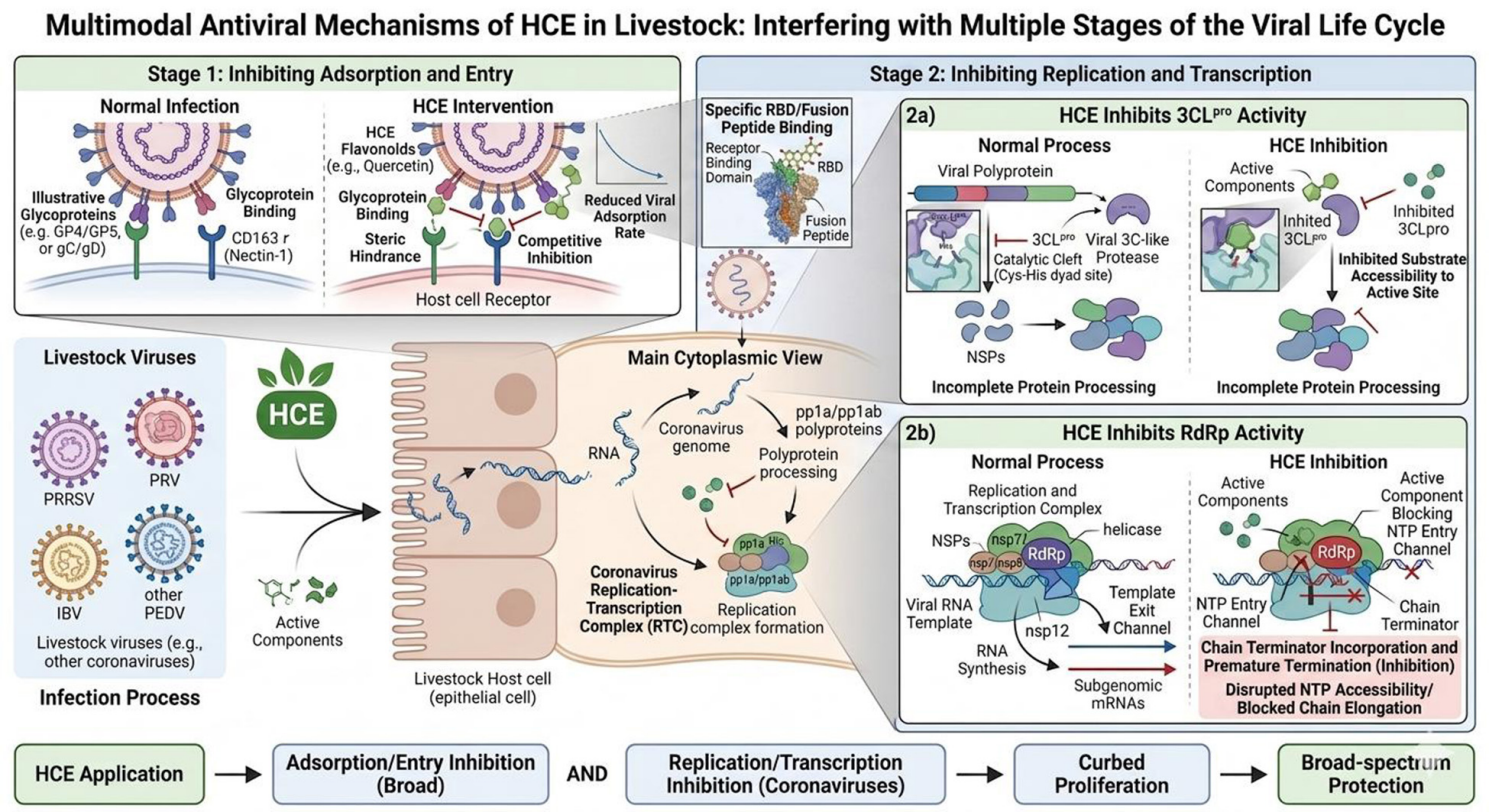
Antiviral mechanism of HCE.

HCE exhibits broad-spectrum inhibitory activity against various viruses detrimental to livestock, with mechanisms involving multiple stages of the viral life cycle (Figure 4). During the viral adsorption and entry stage, flavonoid components in HCE (e.g., quercetin) can bind to glycoproteins on the viral envelope, creating steric hindrance and competitively inhibiting virus binding to host cell surface receptors (36). For instance, in studies on porcine reproductive and respiratory syndrome virus (PRRSV) and pseudorabies virus (PRV), HCE treatment significantly reduced the adsorption rate of viral particles to host cells (37). During the viral replication stage, for coronavirus pathogens such as infectious bronchitis virus (IBV) and porcine epidemic diarrhea virus (PEDV), active components in HCE can specifically inhibit the activity of key enzymes essential for viral replication, such as the 3C-like protease (3CLpro) or RNA-dependent RNA polymerase (RdRp) (38). This enzymatic inhibition directly interferes with viral RNA synthesis and subsequent protein processing, effectively curbing viral proliferation within cells at the transcriptional and replicative levels (39).

## 4 Application of Houttuynia cordata and its extracts in animal husbandry

In ruminant production, HCE effectively improves growth performance and alleviates oxidative stress and inflammatory responses. In Guizhou black goats under summer heat stress, HCE supplementation increased average daily gain (ADG) and the apparent digestibility of nutrients such as dry matter and crude protein. It also enhanced antioxidant capacity by elevating serum levels of glutathione peroxidase (GSH-Px) and total antioxidant capacity (T-AOC), while reducing the expression of pro-inflammatory cytokines (IL-6, IL-8, TNF-α) (8). In studies on lactating dairy cows, dietary HC supplementation significantly mitigated the increase in respiratory rate and rectal temperature induced by heat stress. By altering the fecal microbiome (e.g., increasing *Akkermansia* abundance) and plasma metabolomic profile, it ameliorated redox imbalance and increased milk yield (40). Furthermore, regarding the clinically prevalent issue of bovine mastitis, the core volatile oil component of HC, 2-methylnonyl ketone (MNK), protected bovine mammary epithelial cells from LPS-induced oxidative damage and inflammatory injury by inhibiting the TLR4-NF-κB signaling pathway and activating the Nrf2/HO-1 pathway (41).

Beyond systemic antioxidant effects, HCE significantly modulates rumen fermentation dynamics. Research indicates that HCE can alter the production of volatile fatty acids (VFAs), specifically increasing propionate levels while maintaining a stable NH3-N concentration, which optimizes energy utilization for the host (8). Liu et al. (42) reported that HCE significantly reduced the excretion of intestinal coccidial oocysts in lambs, increased the average daily gain, and enhanced the abundance of beneficial gut microbiota, while also being associated with key metabolites such as 6-deoxyerythronyl aminobenzamide B, thereby demonstrating superior anticoccidial effects and growth-promoting activity. Furthermore, the protective effect of HCE on bovine mastitis is increasingly linked to the “gut-mammary axis”. By stabilizing the intestinal microbiota and reducing the translocation of gut-derived endotoxins (LPS), HCE mitigates systemic inflammatory triggers that lead to blood-milk barrier disruption (8, 30, 39).

In monogastric animal models and production applications, HC and its extracts show unique advantages in antiviral activity and meat quality improvement. Against PRRSV, HC ethanol extract significantly interfered with viral replication *in vitro*. *In vivo*, it reduced viremia by enhancing the expression of interferon regulatory factor 3 (IRF3), demonstrating favorable immunomodulatory and cross-protective effects (43). In meat rabbit production, HCE supplementation not only improved growth performance by upregulating the expression of Nrf2 and antioxidant enzyme genes (CAT, GPX) but also significantly increased the vitamin and polyunsaturated fatty acid content in muscle, optimizing meat traits and volatile flavor profiles (44). Similarly, in poultry studies, dietary HC powder supplementation improved feed conversion ratio (FCR), optimized cecal microbiota, and reduced breast meat fat content in broilers (45). A compound microecological agent containing HC regulated the transcriptome of laying ducks via the “microbiota-gut-ovary axis,” significantly enhancing egg production performance and follicular development quality (46). Meanwhile, HCE exerts significant protective effects in mouse and chicken models of Salmonella infection by downregulating HilA, the central regulator of T3SS-1, thereby inhibiting effector protein secretion and bacterial invasion. This results in marked alleviation of host pathology, reduced intestinal barrier damage, decreased bacterial load in target organs, and modulation of inflammatory responses (47). Relevant experiments in ICR mouse models also confirmed the safety and feasibility of HCE in improving growth performance and increasing the population of intestinal probiotic bacteria (e.g., *Lactobacillus*) (48). In a mouse model of hyperuricemia induced by yeast combined with potassium oxonate, Houttuynia cordata extract dose-dependently reduced serum uric acid and increased urinary uric acid. The underlying mechanisms involve inhibition of hepatic xanthine oxidase activity to decrease uric acid production, as well as promotion of uric acid excretion via downregulation of renal GLUT9 and upregulation of OAT1 and ABCG2 mRNA expression. Additionally, the extract alleviated renal histopathological damage (49).

In the field of aquaculture, HC serves as an environmentally friendly immunostimulant, significantly strengthening the innate immune barriers of aquatic species against viral challenges. Research indicates that feeding common carp (*Cyprinus carpio*) a diet containing 3% HCE significantly upregulated the expression of anti-inflammatory factors (IL-10, TNF-α) in the fish, enhancing their resistance to spring viremia of carp virus (SVCV) (50). In rohu (*Labeo rohita*) fingerlings, supplementation with 10 g/kg of HC leaf ethanol extract significantly increased hemoglobin levels and non-specific immune parameters (e.g., respiratory burst activity and lysozyme activity). It also promoted the expression of IFN-γ and TNF-α genes in the kidney and liver (51). Additionally, studies on Nile tilapia (*Oreochromis niloticus*) confirmed that, while HC powder (FWP) had no direct impact on growth rate, it significantly elevated lysozyme and peroxidase activities in both skin mucus and serum, enhancing the skin mucosal immune defense capacity of the fish (52).

In summary, through the synergistic regulation of key molecular pathways (e.g., Nrf2, NF-κB), optimization of host microbial communities, and reinforcement of innate immune barriers, Houttuynia cordata and its extracts have demonstrated substantial potential as broad-spectrum, efficient, and eco-friendly natural functional feed additives. They show promise in enhancing the growth performance, stress resilience, and disease resistance of ruminants, monogastrics, and aquatic animals.

While HCE has shown consistent benefits, some studies report inconsistent results regarding growth promotion in specific aquatic species, suggesting that its efficacy may be dose-dependent or influenced by baseline stress levels. Furthermore, the poor water solubility of volatile oils remains a barrier to uniform delivery in drinking water systems. We have summarized the research results of HCE on animals in Table 2.

**Table 2:** Systematic evaluation of HCE efficacy on performance, immune status, and production quality across species.

Animal category	Specific species	Performance indicators	Health & immune status	Production quality & microbiota
Ruminants	guizhou black goats, dairy cows, lambs	Increased average daily gain (adg) and dry matter/crude protein digestibility	Alleviated heat stress (respiratory/rectal temp); elevated serum GSH-Px and T-AOC, reduced coccidial oocyst excretion in lambs	Increased milk yield; reduced somatic cell count (scc); increased akkermansia abundance, improved gut microbiota and associated metabolites
Monogastrics	piglets, broilers, laying ducks	Optimized feed conversion ratio (FCR); improved egg production and body weight, in Salmonella infection models: inhibited T3SS-1, reduced intestinal barrier damage	Reduced PRRSV viremia via IRF3; reinforced intestinal tight junctions (ZO-1, Occludin)	Reduced abdominal fat; improved follicular quality; increased lactobacillus population
Specialty/lab	Meat rabbits, ICR mice	Enhanced growth rate and improved nutrient absorption	Upregulated antioxidant genes (Nrf2, CAT, GPX); inhibited apoptosis cascades	Increased muscle PUFAs and vitamins; optimized volatile flavor profiles, attenuated renal histopathological damage in hyperuricemic mice
Aquaculture	Common carp, tilapia, rohu	Improved fingerling growth rate and hemoglobin levels	Enhanced resistance to SVCV; increased respiratory burst and lysozyme activity	Fortified skin mucosal defense; upregulated renal/hepatic IFN-γ and TNF-α

## 5 Challenges and future perspectives

Despite the promising application prospects demonstrated by HCE in laboratory and clinical trials, several technological and industrial bottlenecks must be addressed to achieve large-scale industrial implementation.

### 5.1 Establishment of a standardized quality control system

Herbal extracts commonly face the challenge of “varied efficacy due to different origins.” The volatile oil and flavonoid content of HC can vary greatly depending on the geographical origin, harvesting season, and even plant part used (aerial parts vs. rhizomes) (53). The industry urgently needs to establish quality control standards based on multi-component fingerprinting rather than relying solely on the quantitative detection of a single marker compound (e.g., houttuynin or quercitrin). This is essential to ensure consistent bioactivity across different production batches (54).

### 5.2 Development of novel formulation technologies

Key components in HC volatile oil, such as houttuynin, are chemically highly unstable, decomposing upon exposure to heat and light and being prone to oxidation. The traditional method of direct powder addition leads to substantial loss of active components during feed processing and storage (55). Future research and development should focus on advanced formulation technologies:

Microencapsulation: This technique employs wall materials like cyclodextrins or modified starches to encapsulate thermosensitive components such as volatile oils. It physically isolates them from oxygen, light, and high temperatures, ensures stability in the gastric acidic environment, and enables targeted release of active ingredients in the intestine, thereby improving efficacy and persistence (55).

Nanoemulsions: HCE can be prepared into nano-scale emulsions with particle sizes below 200 nm using methods such as microfluidization, ultrasonication, or phase inversion temperature (56). This technology not only significantly enhances the physicochemical stability of HCE within complex feed matrices but also improves its water dispersibility and intestinal permeability. Consequently, it substantially increases bioavailability and target tissue delivery efficiency (57).

### 5.3 In-depth research on synergistic blends (synbiotics)

The use of HCE alone may have limited efficacy against complex infections. Formulating blends of HCE with probiotics, organic acids, or other plant essential oils (e.g., cinnamaldehyde, thymol) often yields synergistic effects where “1+1 > 2” (58). For example, the combination of HCE with acid-producing bacteria may help construct a more robust intestinal barrier, representing a mainstream direction for future functional feed additive formulations (8).

### 5.4 Safety and toxicology of Houttuynia cordata extract

Although HCE exhibits significant efficacy as a phytogenic feed additive, a balanced assessment of its safety profile is essential for its application in animal production systems. The safety of HCE is extract- and dose-dependent, with quality control playing a decisive role.

Dose-dependent adverse effects. Toxicological studies have established the safety margins of HCE. Acute toxicity evaluation of HCE showed no potential toxicity in rats at a dose of 50 g/kg body weight; subacute administration in rabbits at up to 1.5 g/kg/day also revealed no adverse effects (59). However, higher doses may induce toxicity signals. In a subacute toxicity study in rats, a NOAEL (no-observed-adverse-effect level) was determined at a lower daily dose, while higher doses produced signs of toxicity. Additionally, sodium houttuyfonate (a stable derivative of the volatile oil component houttuynin) has been associated with potential liver damage, gastrointestinal discomfort, and allergic reactions upon long-term use or high-dose exposure (60, 61). In ruminants and monogastric animals, no adverse effects have been reported with HCE supplementation. Nevertheless, establishing a multi-component quality control system and conducting regular monitoring of ALs content are crucial for ensuring batch-to-batch safety and regulatory compliance. In the post-antibiotic era, continued safety surveillance and species-specific dose optimization remain necessary to facilitate broader industrial adoption.

## 6 Conclusion and future perspectives

Existing evidence confirms that HCE is a high-value, green alternative to AGPs in modern animal agriculture. Its multi-target efficacy is driven by a synergistic interplay between volatile oils, flavonoids, and polysaccharides, which systemically modulate the host via the Nrf2/HO-1 antioxidant axis and the TLR4/NF-κB inflammatory axis. HCE has demonstrated robust potential in improving growth performance across ruminant, monogastric, and aquatic systems while simultaneously bolstering disease resistance and meat quality.

To facilitate large-scale industrial adoption, future research must transition from descriptive studies to precision application. Key priorities include:

Standardization: Developing multi-component fingerprinting to ensure consistent quality across different geographical origins.

Stability: Utilizing microencapsulation and nano-emulsification to protect volatile components like houttuynin from degradation during feed processing.

Synergy: Exploring “synbiotic” formulations with probiotics or organic acids to maximize intestinal health benefits.

Ultimately, HCE is poised to become a cornerstone of sustainable, antibiotic-free production systems.
